# MHC Class II Protein Turnover *In vivo* and Its Relevance for Autoimmunity in Non-Obese Diabetic Mice

**DOI:** 10.3389/fimmu.2013.00399

**Published:** 2013-11-25

**Authors:** Alessandra De Riva, Robert Busch

**Affiliations:** ^1^Department of Medicine, University of Cambridge, Cambridge, UK

**Keywords:** autoimmune pathogenesis, type 1 diabetes mellitus, major histocompatibility complex class II, antigen-presenting cell, protein turnover, mass spectrometry

## Abstract

Major histocompatibility complex class II (MHCII) proteins are loaded with endosomal peptides and reside at the surface of antigen-presenting cells (APCs) for a time before being degraded. *In vitro*, MHCII protein levels and turnover are affected by peptide loading and by rates of ubiquitin-dependent internalization from the cell surface, which is in turn affected by APC type and activation state. Prior work suggested that fast turnover of disease-associated MHCII alleles may contribute to autoimmunity. We recently developed novel stable isotope tracer techniques to test this hypothesis *in vivo*. In non-obese diabetic (NOD) mice, a model of type 1 diabetes (T1D), MHCII turnover was affected by APC type, but unaffected by disease-associated structural polymorphism. Differences in MHCII turnover were observed between NOD colonies with high and low T1D incidence, but fast turnover was dispensable for autoimmunity. Moreover, NOD mice with gene knockouts of peptide loading cofactors do not develop T1D. Thus, fast turnover does not appear pathogenic, and conventional antigen presentation is critical for autoimmunity in NOD mice. However, shared environmental factors may underpin colony differences in MHCII protein turnover, immune regulation, and pathogenesis.

## Introduction

Major histocompatibility complex class II (MHCII) molecules present peptides at cell surfaces for recognition by CD4^+^ T lymphocytes. They shape a functional, self-tolerant T-cell repertoire *via* positive and negative thymocyte selection and support peripheral CD4^+^ T-cell homeostasis (1). Presentation of pathogen-derived peptides activates specific CD4 T cells, initiating adaptive immunity (2). Extensive polymorphism in the peptide-binding groove, shaping the presented peptide repertoire, is a key characteristic of MHCII proteins (3). These polymorphisms also control susceptibility to autoimmune diseases.

The regulation of MHCII protein expression is critical to these functions. Here, we discuss MHCII protein turnover, a critical but poorly understood determinant of steady-state expression. We describe recent methodological advances in measuring MHCII protein turnover *in vivo*, which enabled us to examine the postulated role of MHCII protein turnover in autoimmune pathogenesis in a mouse model.

## Functional Significance of MHCII Protein Turnover

Major histocompatibility complex class II proteins are expressed constitutively by antigen-presenting cells [APCs: B cells, dendritic cells (DCs), macrophages, thymic epithelial cells] and inducibly by other cell types. MHCII gene transcription is controlled by the master regulator, class II transactivator (CIITA) (4). Post-translational regulation fine-tunes MHCII expression levels and determines antigen persistence. For example, in DCs, CIITA, and MHCII gene transcription are shut down following activation by bacterial lipopolysaccharide, yet MHCII surface levels rise, because MHCII protein degradation is also shut down (5). As a result, long-lived MHCII/peptide complexes are able to survive during DC migration to lymph nodes (6), where they provide persistent stimuli for CD4^+^ T-cell priming (7). In other APC types, cytokine regulation of MHCII turnover also affects expression (8, 9), and stimuli can increase turnover, rather than shutting it down.

## Determinants of MHCII Protein Turnover

Major histocompatibility complex class II degradation in non-activated DCs begins with internalization from the cell surface, which is triggered by ubiquitination of the cytoplasmic tail of the MHCII β chain (10) by the ubiquitin E3 ligase, membrane-associated RING-CH 1 (MARCH-1) (11, 12). The α chain is also ubiquitinated to a small extent (13). When activated by proinflammatory stimuli, conventional DCs downregulate MARCH-1, terminating MHCII protein turnover; in contrast, activated plasmacytoid DCs maintain this process (14). MARCH-1 also mediates cytokine regulation of MHCII turnover in monocytes and B cells (8, 9, 15). Other MARCH family members may be involved, as well (16).

The internalized MHCII molecules are targeted for lysosomal degradation by unknown proteases. Studies using inhibitors suggest a role for cysteine proteases, although the effects are small (17) and MHCII molecules resist proteolysis by many cysteine cathepsins *in vitro* (18). The serine protease, Cathepsin G, cleaves MHCII molecules at a specific membrane-proximal site *in vitro*, but this does not detectably contribute to MHCII degradation in APCs (18).

**Figure 1:**
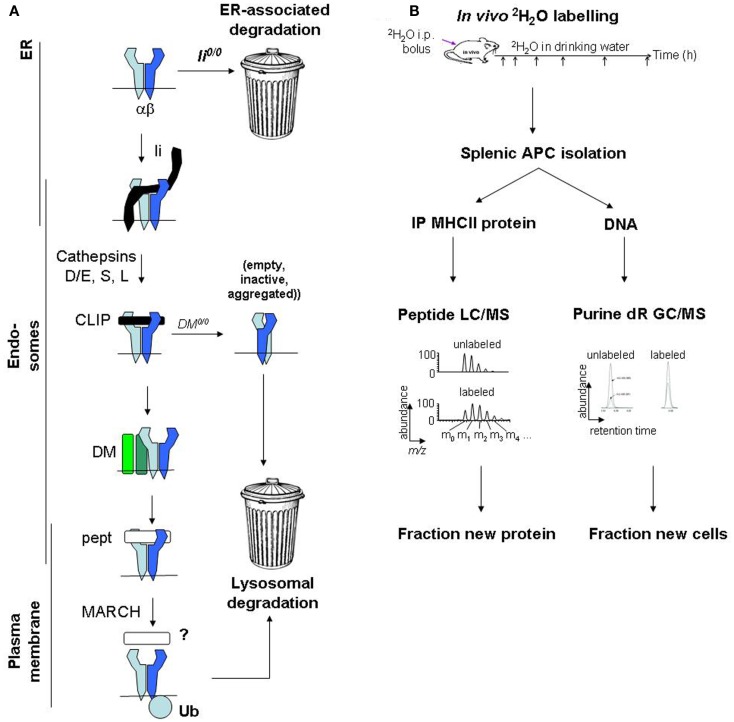
**Major histocompatibility complex class II protein turnover: molecular regulation and mass spectrometric measurement**. **(A)** Regulation of MHCII protein turnover in APCs. **(B)** Work flow for SINEW measurement of MHCII protein turnover in mice.

Major histocompatibility complex class II protein degradation is also influenced by the preceding assembly and maturation steps, which are reviewed elsewhere in this series (Figure 1) (19). Briefly, αβ heterodimers assemble with invariant chain (Ii) in the endoplasmic reticulum (ER) and travel to endosomes. There, Ii is cleaved, leaving class II-associated Ii peptides (CLIP) in the peptide-binding groove, which must be released to enable loading with endosomal peptides. A co-factor, DM (HLA-DM in humans, H2-DM/H2-M in mice), releases CLIP, “edits” peptides to select stable binders, and stabilizes empty MHCII molecules (“chaperoning”). Finally, MHCII/peptide complexes travel from endosomes to the plasma membrane. Defects in these maturation steps accelerate MHCII protein degradation, sometimes by use of alternative degradation pathways. In Ii-deficient cells, a significant proportion of MHCII molecules are retained in the ER and degraded (20), presumably by the proteasome following retrotranslocation to the cytosol. In DM-deficient cells, the turnover of some MHCII alleles in post-Golgi compartments is accelerated, likely because MHCII acquire loosely bound peptides and are not chaperoned following spontaneous CLIP release (21). Moreover, peptide occupancy appears to protect MHCII molecules from degradation even in normal APCs: exposure of splenocytes to high doses of exogenous antigen protects MHCII molecules from degradation, increases their resistance to disruptive detergents (SDS), and raises their surface levels (22).

Many details of these mechanisms remain ill-defined. Moreover, while polymorphisms may affect MHCII fate in DM-deficient cells, *via* their impact on CLIP affinity, it is unclear to what extent MHCII allelic variants may differ in their turnover rates in normal APCs.

## Could Accelerated MHCII Protein Turnover Promote Autoimmunity?

A role for MHCII protein turnover in autoimmunity was first proposed in non-obese diabetic (NOD) mice, which develop autoimmune beta-islet cell destruction leading to type 1 diabetes (T1D) under complex genetic, environmental, and developmental control. The sole expressed MHCII gene product, H2-A^g7^, is critical for T1D development [recently reviewed in detail by Busch et al. (23)]. A^g7^ has a unique β chain, differing by 17 amino acids from Aβ^d^; its α chain is identical to Aα^d^. A^g7^ shares structural features with HLA-DQ alleles associated with T1D in humans, including a key non-Asp57β polymorphism, affecting the P9 specificity pocket.

**Figure 2:**
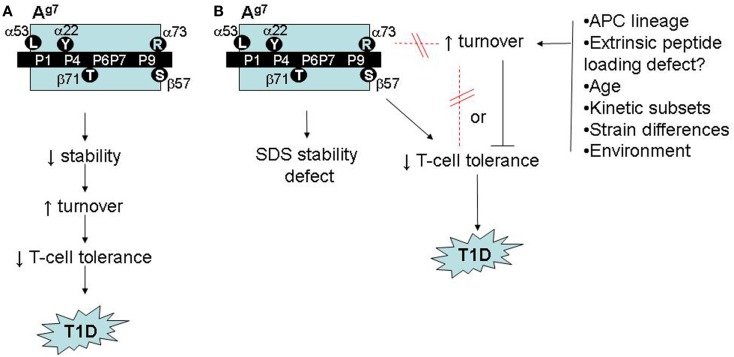
**Possible roles of accelerated A^g7^ protein turnover in autoimmune pathogenesis in NOD mice**. **(A)** Model adapted from (26) and (27), linking A^g7^ polymorphism to intrinsic stability defects, accelerated turnover, impaired T-cell tolerance, and increased risk of autoimmunity. **(B)** Revised model, based on (53), showing accelerated A^g7^ turnover *in vivo* to be regulated, independently of structural polymorphisms and SDS instability, by unknown environmental factors, with either a neutral or inhibitory role in autoimmune pathogenesis.

One reason to suspect that A^g7^ polymorphisms confer a broader structural deficit is that A^g7^-restricted autoreactivity in NOD mice is not islet-specific. Crossing NOD mice with KRN T-cell receptor transgenic mice serendipitously creates reactivity to a ubiquitously expressed, A^g7^-presented self peptide, triggering autoimmune arthritis (24). Moreover, NOD mice exhibit spontaneous autoreactivity (25), and immunization with foreign antigens elicits A^g7^-restricted bystander responses to self peptides (26). Numerous unusual biochemical features of A^g7^ might contribute to the tolerance deficits (Figure 2A):

- Numerous peptides bind weakly to, or dissociate rapidly from, A^g7^ (27–30). Peptide binding to A^g7^ may be unusually promiscuous (31).
- A^g7^ has low CLIP affinity at endosomal pH (32). Paradoxically, CLIP levels on NOD splenocytes are high (33), suggesting inefficient DM editing *in vivo*, which could diminish the levels or stability of A^g7^ complexes with self peptides and accelerate turnover (21, 34). The reason is unclear, as A^g7^ seems to interact productively with DM in transfectants (35). Unstable peptide binding may be determined extrinsically, rather than by A^g7^ structural polymorphism.
- One study has shown unusually fast turnover of A^g7^ in NOD splenocytes *in vitro* (27).
- A^g7^ heterodimers dissociate at room temperature in the presence of SDS detergent (27), more so than other H-2A alleles. This feature is shared by human DQ T1D risk alleles (36). Although its significance is unclear, SDS instability could correlate with some of the other biochemical features described above.

Other studies, however, questioned the relevance of these findings to pathogenesis. The SDS instability of A^g7^ may represent the lower range of normal variation (37). Not all studies found defects in peptide binding selectivity or life span of A^g7^ (37, 38). Studies of soluble recombinant A^g7^ provided no evidence for stability defects (39, 40). Lastly, the biochemistry of A^g7^ differs from that of the homologous human HLA-DQ T1D risk alleles, DQ2 and DQ8, in key details (e.g., the mechanism of CLIP retention (41), so these details may not be critical for pathogenesis.

Peptide-specific mechanisms for A^g7^-restricted autoreactivity have also been proposed. A^g7^ selects peptides similar to those binding human DQ T1D risk alleles (38). Neo-self determinants may be generated by post-translational modification of peptides by transglutaminases, resulting in increased binding to A^g7^ (42), analogous to mechanisms explaining DQ associations with celiac disease in humans (23). Both peptide-dependent and -independent mechanisms could contribute to A^g7^ associations with autoimmunity.

## *In vivo* Measurements of Protein Turnover: A Methodological Excursion

Given these uncertainties, we re-examined whether A^g7^ molecules exhibit unusually fast turnover in NOD mice, and what this meant for autoimmunity. We also quantified the baseline turnover of MHCII molecules in APCs of normal mice for the first time. This required the development of novel methods for measuring MHCII protein turnover *in vivo*.

Protein turnover is measured using tracers, which are biosynthetically incorporated into newly synthesized proteins and persist until the protein is degraded. In cell culture, newly synthesized proteins may be tagged with radiolabeled amino acids (e.g., [^35^S]-Met/Cys); during a subsequent chase, the fate of the labeled proteins is tracked by autoradiography (43). This approach is powerful but difficult to use *in vivo*, because substantial amounts of radioactivity would be required, even in small-animal models.

Stable isotope tracers, lacking radiation hazards, are easier to use *in vivo*. “SILAC” (stable isotope labeling of amino acids in cell culture), used for kinetics rather than quantitation, is one variant of this approach (44). Arg and Lys in culture media are replaced with all-[^13^C]-Arg/Lys, and their incorporation into newly synthesized proteins is quantified by mass spectrometric analysis of tryptic digests. The relative abundances of unlabeled and labeled peptides define the proportion of molecules that were synthesized during the labeling interval and survived until analysis (“fractional protein synthesis”). The approach has been adapted for *in vivo* use (45). However, label entry into amino acyl-tRNAs used for protein synthesis is not fully understood; pools of unlabeled amino acids may persist in animals even after long-term labeling.

An alternative strategy uses deuterated water (^2^H_2_O) as a biosynthetic label. ^2^H_2_O labeling is simple and inexpensive to perform in cell culture and *in vivo* (46). At levels used for metabolic labeling (1–5% in body water), ^2^H_2_O does not interfere with normal physiology. It rapidly equilibrates throughout the body following administration. Protons from water are used primarily in the (comparatively rapid) synthesis of non-essential amino acids, such as Ala, by cells (47). For proteins with turnover rates of a few hours or longer, protein synthesis is the rate-limiting step for ^2^H label incorporation from ^2^H_2_O (48, 49). This approach was named “SINEW” (stable isotope labeling of non-essential amino acids with heavy water). Like SILAC, SINEW tracks the proportions of unlabeled to labeled tryptic peptides by mass spectrometry. Although these species are less well resolved in SINEW experiments, precise measurements of fractional protein synthesis rates are possible (50–52).

New protein is synthesized not only to replace protein lost to turnover, but also to maintain protein levels as cells divide. For cell-associated proteins with slow turnover rates, the proliferative contribution can be considerable. During SINEW experiments, this contribution can be quantified accurately by measuring ^2^H incorporation from ^2^H_2_O into newly synthesized DNA (46). SINEW was therefore used to explore MHCII protein turnover (53).

## Extrinsic Regulation of MHCII Protein Turnover *In vivo*

To quantify A^g7^ protein turnover *in vivo* (53), we labeled young female prediabetic NOD mice with ^2^H_2_O; BALB/c mice (A^d^, E^d^) were used as controls. MHCII molecules were immunoprecipitated from splenic B cells and DCs (which together harbor most splenic MHCII molecules), resolved on SDS gels, digested with trypsin, and analyzed by mass spectrometry. MHCII-derived peptides were identified, and changes in their mass distributions from ^2^H_2_O labeling were used to quantify fractional protein synthesis (Figure 1B). MHCII protein synthesis greatly exceeded the rate required to support the turnover of the APCs themselves (quantified by analysis of ^2^H incorporation into DNA). Thus, fractional protein synthesis reflected MHCII protein turnover rates.

The turnover rates of MHCII proteins *in vivo* proved to be markedly affected by the cellular microenvironment: turnover half-lives (*t*_1/2_) were ≈10–12 h in B cells but twice as fast (*t*_1/2_ ≈5–6 h) in DCs (53). Thus, turnover rates measured in unseparated splenocytes (27) might be somewhat confounded by differences in APC composition, because NOD splenocytes contained a greater proportion of DCs, relative to B cells, than BALB/c splenocytes. Other APC types or locations of potential relevance to pathogenesis were not analyzed here.

Given that inflammatory stimuli influence MHCII protein turnover rates (5), it should be noted that these experiments were performed in specific pathogen-free mice, which exhibited no detectable activation of splenic APCs; conceivably, altered MHCII protein turnover in a few activated APCs might be relevant to pathogenesis. The difference in MHCII half-lives between APC types may reflect the greater extent of ubiquitination and internalization of MHCII molecules in DCs (54).

We also examined A^g7^ turnover in transfected M12 B lymphoma cells (50, 53). These cells doubled approximately every 27 h and produced new A^g7^ molecules at the same rate. Thus, A^g7^ protein synthesis was attributable to cell growth, with only a small contribution from turnover. The implied long A^g7^ half-life (>2 days) argues against an intrinsic stability deficit of this allele and confirms a substantial impact of the cellular microenvironment on MHCII turnover rates. These findings also indicated that physiological turnover rates are not necessarily recapitulated in model cell lines.

Within each cell type, MHCII alleles apparently differed in their dynamics (53): the half-life of A^g7^ molecules in NOD B cells was significantly shorter than that of A^d^ in BALB/c B cells, albeit by only a small amount (13%). However, kinetic modeling indicated that that A^g7^ turnover was heterogeneous: its ^2^H_2_O labeling curve was significantly better explained by a mixture of long- and short-lived molecules than by a uniform half-life.

The presence in NOD (but not BALB/c) B cells of some H2-A molecules with faster turnover could reflect strain differences in the cellular microenvironment. To address this, we bred (NOD × BALB/c) F1 mice, expressing both H2-A molecules in the same cells. In F1 APCs, the turnover of A^d^ and A^g7^ was indistinguishable (53), so the cellular microenvironment is decisive. The fast-turnover component may be attributable to inefficient peptide loading in NOD splenocytes, although this is not the only possible explanation.

## Accelerated A^g7^ Turnover is Dispensable for Autoimmunity

Several observations suggested that fast-turnover A^g7^ molecules do not correlate closely with T1D development (53). A^g7^ turnover is indistinguishable between B cells from male and female NOD mice, yet T1D incidence is lower in males. Moreover, the fast-turnover subpopulation detectable in young female NOD mice becomes undetectable at 12 weeks of age, prior to the onset of T1D.

More compellingly, we initially observed the fast-turnover A^g7^ molecules in one colony of NOD mice, which exhibited a relatively low incidence of T1D (22% diabetic females at 30 weeks of age). When we repeated the SINEW experiments in a second NOD colony, which develops a much higher T1D incidence (>80% at 30 weeks in females), its A^g7^ molecules showed no accelerated turnover (53). Thus, fast A^g7^ turnover appears dispensable for T1D development (Figure 2B).

This conclusion might seem disappointing, yet our findings may harbor a critical clue. Worldwide, NOD colonies differ widely in their disease incidence as a result of unknown environmental variables (55); our two colonies represent extremes on this spectrum. The environmental regulator(s) in our colony remain unknown but appear to be novel. Our results suggest, however, that key environmental influences may be sensed by B cells in secondary lymphoid organs, triggering changes in the dynamics of antigen presentation (Figure 2B). Dissection of these mechanisms may yield novel insights into mechanisms of gut microbe/immune interactions in autoimmunity.

## Normal MHCII Peptide Loading Required for Autoimmunity in NOD Mice

Intact MHCII peptide loading pathways are critical to T1D pathogenesis in NOD mice. Invariant (Ii) chain-deficient NOD mice express reduced levels of A^g7^ and are protected from T1D (56). Their effector CD4^+^ T cells have islet autoreactivity similar to Ii-sufficient NOD mice, but the frequency of regulatory T cells (Tregs) is increased (57). Similarly, DM-deficient NOD mice show reduced A^g7^ surface expression and are protected from T1D development (58). These mice also have increased Treg frequencies but, unlike Ii-null NOD mice, do not develop autoreactive effector T cells, suggesting that presentation of islet autoantigens requires peptide editing. A more subtle defect was engineered into DCs of NOD mice by transgenic expression of HLA-DO, an inhibitor of DM, under control of the CD11c promoter (59). These mice also failed to develop T1D, indicating an important role of normal MHCII peptide editing in DCs during T1D pathogenesis. Because defects in MHCII peptide loading pathways accelerate A^g7^ protein turnover (21, 34), the findings are consistent with our observations that fast-turnover A^g7^ molecules are dispensable for autoimmunity (53). Indeed, the increased Treg frequencies caused by peptide loading defects raise the possibility that short-lived A^g7^ might be protective.

## Conclusion

Turnover rates of MHCII molecules *in vivo* depend strongly on APC type and are affected by environmental factors, but not substantially by the MHCII structural polymorphisms we have examined. Whether MHC protein turnover is generally insensitive to structural polymorphism in normal APCs is an interesting question, given the allelic differences previously observed in the presence of peptide loading defects. Work is ongoing to extend these observations, explore their functional and evolutionary significance, and examine the underlying molecular mechanisms. Accelerated A^g7^ turnover in NOD spleens affects only a subset of A^g7^ molecules and does not represent an intrinsic stability defect associated with this risk allele. Moreover, this phenotype is dispensable for autoimmune pathogenesis, and might even counteract autoimmunity (though the latter possibility will be difficult to prove). Surprisingly, colony differences in A^g7^ turnover reveal novel potential connections between environmental regulators, B cells, and autoimmune pathogenesis, which are now being explored. Lastly, SINEW, used here to quantify MHCII protein turnover, has important advantages in quantifying the turnover of cell-associated proteins. SINEW shows promise for probing genotype/phenotype associations and normal and pathological immune responses.

## Conflict of Interest Statement

Robert Busch holds stock in KineMed Inc., a biopharmaceutical company with related commercial interests and intellectual property, and is a former employee and consultant for this company. The other co-author declares that the research was conducted in the absence of any commercial or financial relationships that could be construed as a potential conflict of interest.
